# Why increase in telework may have affected employee well-being during the COVID-19 pandemic? The role of work and non-work life domains

**DOI:** 10.1007/s12144-023-04250-8

**Published:** 2023-01-26

**Authors:** Janne Kaltiainen, Jari J. Hakanen

**Affiliations:** Work Ability and Working Careers, Finnish Institute of Occupational Health, P.O. Box 40, FI-00032, Työterveyslaitos Helsinki, Finland

**Keywords:** Telework, Employee well-being and engagement, Work-nonwork/work-family, COVID-19, Latent change score analysis, Job design

## Abstract

Given that millions of employees switched to mandatory telework during COVID-19, and as teleworking practices are likely to continue, it is essential to understand the potential impact of mandatory and non-flexible teleworking practices on employee well-being. Drawing on Conservation of Resources theory, we find support for resource gains as increase in job control mediates the link between increases in teleworking and improvements in well-being (increases in work engagement and decreases in burnout and job boredom). Conversely, the findings indicate resource losses as loss of social support and higher work-non-work interference explain the deterioration in well-being for those whose teleworking increased since the COVID-19 outbreak. By coupling role depletion and role enrichment theories, we find that employees who had children living at home experienced greater work-non-work interference due to increased teleworking. However, having children also buffered the negative impact of work-non-work interference on well-being. The findings are based on a Finnish population sample collected three months before and after the COVID-19 outbreak in March 2020 (*N* = 996 of matched respondents) and latent change score analyses of within-person changes. Our results provide new insights regarding the potential impact of teleworking on employee well-being in a context where teleworking is not flexible and self-selected by the employees. Notably, our results draw bridges (rather than burn them) between role depletion and enrichment frameworks as having children may simultaneously be “a burden” and “a gift”.

**Supplementary information:**

The online version contains supplementary material available at 10.1007/s12144-023-04250-8.

As a result of the COVID-19 outbreak, millions of employees were forced to switch to teleworking from home and the trend of increasing telework likely lasts beyond the pandemic (Eurofound, 2020; Milasi et al., 2020). At the same time, the global pandemic has threatened the well-being of millions of employees (Restubog et al., 2020). Given that employees’ psychological well-being is associated with numerous outcomes at the individual, organizational, and societal levels (e.g., performance, absenteeism, psychological and physical health; Bakker et al., 2014) it is essential to illuminate to what extent and why forced teleworking may impact well-being and thus provide means to promote well-being in such contexts.

The switch from working at the workplace to teleworking from home represents a substantial change, which is likely to alter the characteristics of work and non-work domains of life (Kramer & Kramer, 2020). Such characteristics may at the same time represent resources (e.g., autonomy at work, time spent with children) or threaten them (e.g., work-life imbalance) both at work and home (Golden, 2006; Hobfoll, 1989). This in turn may explain whether teleworkers’ well-being improves or deteriorates over time (Allen et al., 2003; Gajendran & Harrison, 2007). Notably, as teleworking during the COVID-19 pandemic was not similarly self-selected in terms of frequency and place as before, we provide new insights that cannot be derived from existing research on voluntary teleworking.

In this study, we examine why increase in teleworking during COVID-19 is associated with changes in work-related well-being, namely its positive (work engagement) and negative (burnout and job boredom) dimensions. Whereas work engagement is a positive-motivational state characterized by vigor, dedication, and absorption (Schaufeli et al., 2017), burnout is a state of exhaustion and cynicism that is coupled with impaired cognitive functioning and loss of emotional control at work (Schaufeli et al., 2020). Job boredom in turn represents a negative affective-motivation state characterized by low arousal, mind-wandering, and perceptions of slow passage of time (Reijseger et al., 2013). We examine to what extent job control, social support, and work-non-work interference explain the impact of increase in teleworking during COVID-19 on changes in multiple types of employee well-being. To illuminate further the dynamics between work and non-work domains, we study the potential benefits and disadvantages of having children who live at home as a booster and a mitigator for the hypothesized paths regarding work-non-work interference. The hypotheses are tested in a two-wave sample of matched respondents (*N* = 996) collected three months before and after the COVID-19 outbreak. By this, we provide the following contributions.

First, we illustrate how the processes of both accumulation and loss of resources occur because of increases in telework during COVID-19. We draw from Conservation of Resources theory (COR; Hobfoll, 1989) and examine teleworking during the COVID-19 outbreak, which as a context differs from most of the previous teleworking research (e.g., Kaluza & van Dick, 2022). As elaborated by Kniffin et al. (2021): “A key difference, though, is that work from home was previously often responsive to employee preferences but COVID-19 forced many into mandatory work from home, making it difficult to generalize prior findings” (p. 65). While our decision to examine job control, social support, and work-non-work-interference as the mediator mechanisms is guided by the existing teleworking literature from the pre-COVID-19 era (Allen et al., 2003; Gajendran & Harrison, 2007), we show whether these underlying processes differ when employees cannot similarly choose whether to telework or not. Coupled with the study design examining within-person changes in well-being over time, we provide essential knowledge regarding the potential impact of forced teleworking, which may occur also after the pandemic, and how to effectively promote well-being in such work arrangements.

Second, we contrast and build bridges between two opposing theoretical views in work-non-work interface literature and expand the notions drawn from COR theory (Hobfoll, 1989) with these frameworks. Whereas the role strain perspective (Greenhaus & Beutell, 1985) postulates the downside of having multiple roles such as being a parent and having children, contrastingly role enhancement theory (Rothbard, 2001) posits that multiple roles have a positive and enriching impact. In doing so, we expand the current theoretical understanding of resources in COR theory (Hobfoll, 1989) as we show whether having children is likely to act either as a demanding condition promoting resource loss (accentuating the relationship from increases in telework to higher work-non-work interference) or as a resource mitigating the negative effects of losing other resources (mitigating the relationships from higher work-non-work interference to deterioration of employee well-being) or both. For fostering mental health it is important to learn who are most at risk when working from home, and what are the mechanisms that facilitate well-being.

Third, by examining multiple dimensions of well-being at work rather than measuring only either positive or negative aspects, we provide a more nuanced and holistic understanding regarding the impact of teleworking on employee well-being. For instance, certain characteristics of work and non-work may be more focal in explaining the effects of telework on work engagement than on burnout or job boredom (Taris & Schaufeli, 2018). By this, we add to research that has increasingly applied COR theory to examine also the positive states of well-being (Hobfoll et al., 2018) and provide a more holistic understanding regarding the well-being impact of resource loss and gain. As a practical contribution, we show whether an increase in telework may change employee well-being and how wide-ranging such changes are. Despite the growing understanding of the detrimental effects of job boredom, this negative state of well-being and its antecedents remain understudied, especially so in the context of telework.

## Accumulation and loss of resources as the mechanism between changes in telework and employee well-being

According to COR theory (Hobfoll, 1989), resources represent things, states, or conditions that people value either by themselves or as means to obtain valued objectives. Resources facilitate well-being as they are beneficial for coping in challenging environments and combating the harmful effects of stress. As resources are affected by one’s environment, changes in the environment may produce losses or gains in resources, for instance, by hindering access to or threatening existing resources (Hobfoll, 1989, 2002). As elaborated by Hobfoll et al. (2018): “Environments and contexts create fertile or infertile ground for creation, maintenance, and limitation of resources” (p. 107). As an increase in telework represents such a change in the environment, based on COR theory we expect that whether such change benefits or harms well-being depends on whether it leads to accumulation or loss of resources. For this, we examine the key three mediator mechanisms established in existing pre-COVID-19 era teleworking literature – job control, social support, and work-non-work interference as elaborated below.

While people in general strive to maintain and acquire beneficial resources (Hobfoll, 1989), we extend this theoretical understanding by showing what happens when the circumstances dictate employees’ work arrangements. Specifically, whereas choosing to telework according to one’s preferences may be more likely to accumulate resources as in such context individuals are free to choose work arrangements that benefit them, change in teleworking during the first months of the COVID-19 outbreak was not similarly self-selected. Thus, such increase in teleworking is more likely to represent an ambiguous event which is more prone to lead to both gains and losses in resources (Hobfoll, 1989). As the impact of loss and gain processes may differ depending on the examined well-being outcome (Bakker & Demerouti, 2014) it is also important to simultaneously examine both positive and negative well-being dimensions (see also Debus et al., 2019).

### The role of job control and social support

In the context of work, one valuable resource for one’s well-being is job control as it satisfies the need for autonomy, one of the basic psychological needs (Halbesleben et al., 2014). Following the classic Demand-Control model by Karasek et al. (1998), we use the concept of job control that includes both, autonomy and learning at work. Telework, before and during COVID-19, is typically associated with separation, spatially and psychologically, from office routines and managerial oversight (Gajendran & Harrison, 2007; Kniffin et al., 2021; Stoker et al., 2021). For many, this results in greater autonomy in conducting work tasks, scheduling working time, and prioritizing one’s work tasks (Allen et al., 2015) thus increasing resources that are beneficial for one’s well-being (Hobfoll, 1989). Accordingly, telecommuting is associated with higher autonomy before (for reviews, see Allen et al., 2015; Charalampous et al., 2019; Gajendran & Harrison, 2007) and during the COVID-19 outbreak (Giauque et al., 2022). Furthermore, an increase in telework likely facilitates learning new ways of working that are beneficial for adapting to a new work setting such as home (Rodríguez-Modroño & López-Igual, 2021). Potentially, the distance from the support network at the workplace may also necessitate and thus facilitate more autonomous learning while teleworking. Whether the increase in teleworking is self-selected or not is not likely to impact the subsequent change in one’s job control as job control represents experiences of learning and decision latitude, which both are similarly facilitated by the change in environment (new environment facilitating learning and separation from others facilitating autonomy). The COVID-19 outbreak may even strengthen the salience of teleworker’s job control as it may appear as the life domain that an individual still has control over during substantial general uncertainty (Becker et al., 2022).

According to the theoretical postulation of job control being a valuable resource (Halbesleben et al., 2014), job control is associated with higher work engagement, and lower burnout and job boredom (Bakker et al., 2014; Guglielmi et al., 2013; Reijseger et al., 2013). By increasing stimulation at work via choosing tasks more autonomously, having more decision latitude, and learning at work, job control is likely to promote positive motivational states characterized by high levels of motivation, energy, and enthusiasm, such as work engagement (Bakker & Demerouti, 2014). Similarly, job control is also expected to combat burnout, which is associated with a loss of exerting control at work (Maslach et al., 2001), and job boredom, which is associated with a lack of stimulation and challenges at work (Harju & Hakanen, 2016). We therefore expect that increase in teleworking during COVID-19 will foster job control, representing a resource gain, which mediates the impact of increases in teleworking and improvements in employee well-being based on COR theory (Hobfoll, 1989):

#### Hypothesis 1

*Increase in telework during COVID-19 is associated with* ***(a)*** *increases in work engagement and decreases in* ***(b)*** *burnout and* ***(c)*** *job boredom via increase in job control.*

Whereas moving to telework typically decreases the amount of interaction at work, it does so especially for face-to-face interactions which represent the richest medium for communication in terms of media richness and social presence (Daft & Lengel, 1986). Furthermore, those who telework are ‘out-of-sight’ and thus more likely to be also ‘out-of-mind’ (Kossek et al., 2015). Therefore, teleworking is likely to inhibit relationships with co-workers and supervisors (Golden, 2007; Golden et al., 2008) thus leading to less support from co-workers and supervisors (Allen et al., 2003). We expect this is especially so during times of physical distancing and prolonged mandatory work from home (Kniffin et al., 2021). Social support is a resource that emerges from the social environment and it may include receiving assistance, feedback, appreciation, empathy, caring, and advice from others, such as from co-workers and supervisors (Peeters et al., 1995). A change in the environment that diminishes social interaction is likely to lead to a loss of this valuable social resource which subsequently decreases employee well-being (Hobfoll, 2002). Accordingly, teleworking is associated with isolation and lower social support at work (for reviews, see Allen et al., 2015; Charalampous et al., 2019).

As postulated in COR theory, depletion of resources is likely to lead to decreases in well-being (Hobfoll, 1989). This is because due to resource loss, such as favorable conditions, employees do not anymore have the same means to cope with stressors thus increasing the risk of burnout (Bakker & Costa, 2014). One such essential resource at work is social support (Halbesleben et al., 2014) which as a positive job characteristic also promotes positive affective states, employee functioning, and intrinsic motivation, and thus fosters work engagement (Bakker & Demerouti, 2014). Having social interactions and support from coworkers and supervisors is likely to diversify one’s work by representing a stimulus and thus combat job boredom, which is associated with a lack of stimulation and a monotonous work environment (Loukidou et al., 2009). Accordingly, social support is associated with higher work engagement, and lower burnout and job boredom (Bakker et al., 2014; Reijseger et al., 2013). Given the theorizing and argumentation above, we expect the loss of social support as a loss of resource to mediate the impact of increase in teleworking on deterioration of employee well-being based on COR theory (Hobfoll, 1989):

#### Hypothesis 2

*Increase in telework during COVID-19 is associated with* ***(a)*** *decreases in work engagement and increases in* ***(b)*** *burnout and* ***(c)*** *job boredom via loss of social support at work.*

### The role of work and non-work life domains

To hypothesize the impact of teleworking on higher work-non-work interference during COVID-19, we synthesize the notions of COR theory with the role boundary frameworks (e.g., Clark 2000).^1^ Specifically, teleworking introduces higher permeability of the boundaries of different life domains (Gajendran & Harrison, 2007; Standen et al., 1999). Permeability refers to the degree to which roles occur at the same place and as result, it is easier for one life domain to infer with another potentially leading to role integration and difficulties in maintaining boundaries between life domains (Ashforth et al., 2000; Clark, 2000). This may manifest, for instance, in more frequent interruptions and transitions between work and non-work tasks and roles which may cause higher work-non-work interference (Allen et al., 2021; Delanoeije et al., 2019; Grotto et al., 2021). Whereas teleworking literature from the pre-COVID-19 era has argued that the flexibility and more time control introduced by teleworking, benefits work-life balance, and have found empirical support for this (for reviews, see Allen et al., 2013; Gajendran & Harrison, 2007), such telework flexibility is less likely found during the pandemic. This is because teleworking during COVID-19-related social restrictions is more typically mandatory, high in frequency, prolonged, and takes place at home rather than being self-selected according to one’s preferences, and thus is likely to lead to difficulties in regulating and synchronizing demands between work and non-work (e.g., Blahopoulou et al., 2022; Cho, 2020; Syrek et al., 2021). Recent cross-sectional studies support this notion. Palumbo (2020) and Sandoval-Reyes et al. (2021) found that home-based telecommuting during COVID-19 was negatively associated with work-life balance. Kaduk et al. (2019) found support for involuntary flexible work being associated with high work-family conflict.

Reconciling work and non-work demands simultaneously requires more effort in comparison to more segmented work and non-work life domains and thus also threatens existing resources. Drawing from COR theory, a loss and a threat of losing resources is likely to diminish well-being (Hobfoll, 1989). As a result of work-non work interference, employees need to invest more of their resources into their work role to keep up with their expected performance level and as a consequence experience increases in burnout and decreases in work engagement. Accordingly, interference between work and non-work domains of life is associated with higher burnout (Allen et al., 2000; Amstad et al., 2011; Reichl et al., 2014) and lower work engagement (Opie & Henn, 2013). Given that work-non-work interference is likely to hinder employees’ engagement with tasks that they find stimulating and important (Harju & Hakanen, 2016), work-non-work interference is likely to increase job boredom. Taken together, we expect higher work-non-work interference to represent a resource loss and thus mediating the association between increases in telework and deterioration of employee well-being:

#### Hypothesis 3

*Increase in telework during COVID-19 is associated with* ***(a)*** *decreases in work engagement and increases in* ***(b)*** *burnout and* ***(c)*** *job boredom via higher work-non-work interference.*

While we argue that increases in teleworking are likely associated with higher work-non-work interference, we expect this to be especially so for employees who have children living at home. Here we draw from role depletion literature, which postulates that people have a fixed amount of psychological and physiological resources to expend (i.e., a scarcity hypothesis; Greenhaus & Beutell, 1985). Being an employee and a parent with children at home may cause “a form of inter-role conflict in which the role pressures from the work and family domains are mutually incompatible in some respect” (Greenhaus & Beutell, 1985). The more one domain requires resources, the fewer resources are available to fulfill one’s role in another domain thus leading to conflict between these two domains (Grandey & Cropanzano, 1999). For example, during teleworking from home (e.g., a parent having a Zoom-meeting) the care and attention needed by a child (e.g., a crying child) may disrupt one’s work, and thus lead to higher interference between non-work and work domains. Similarly drawing from COR theory (Hobfoll, 1989), having children may represent a threat to existing resources as children require time and energy to be taken care of, and thus accentuate the experience of work-non-work interference.

Studies have found having children at home to be associated with higher work-non-work interference (Byron, 2005; Michel et al., 2011) and lower telework satisfaction (Blahopoulou et al., 2022). Such effects may occur due to higher time and emotional demands as children require attention and parents may worry about one’s children (Peeters et al., 2005). We believe this is especially so during COVID-19 as there were restrictions in access to schools and daycare. As a result, teleworking employees with children living at home have had to care for and provide help with schoolwork, or even homeschooling, while at the same time managing their work, and thus likely experience higher work-non-work interference (Rudolph et al., 2021). Accordingly, a recent study by Allen et al. (2021) showed that having other people in the same household was associated with lower work-non-work balance amongst employees who transitioned to working from home during COVID-19. Drawing on the theorizing and relevant literature above, we expect having children to accentuate the resource loss process in terms of higher work-non-work interference because of increases in teleworking:

#### Hypothesis 4

*The association between increase in telework during COVID-19 and higher work-non-work interference is stronger amongst those who have children than for those who do not have children living at home.*

In addition to arguing that having children at home is likely to present a challenge during the COVID-19 pandemic in terms of higher work-non-work interference, we expect having children to also benefit employees’ well-being. Here we draw from role enrichment theory (Marks, 1977; Rothbard, 2001), which provides a contrasting view on previously described role deprivation theory. The enrichment argument posits that a greater number of role commitments, such as being a parent in addition to being an employee, provide benefits rather than drain them (e.g. Rothbard, 2001). According to the work-family enrichment theory (Greenhaus & Powell, 2006), there are three reasons why such benefits may occur. First, family and work experiences can have additive effects on well-being. Second, participation in both work and family roles can buffer employees from distress in one of the roles. Third, participation in one role can create energy that can be used to enhance experiences in the other role. For instance, the family domain may fulfill different needs (e.g., love and affection) and provide breaks from the work domain and thus benefit well-being (Clark, 2000). This may be especially so for those who have children as parents generally receive affection from their children. Accordingly, studies have found support for the association between having children and better mental health (Angeles, 2010) and also so among teleworkers during COVID-19 (Blahopoulou et al., 2022).

Similarly, drawing from COR theory (Hobfoll, 1989), having children or being a parent may not only require resource investments but also represent a beneficial condition that mitigates negative effects on well-being. Given that the effect of resources may depend on the context (Hobfoll et al., 2018), having children may be especially important for one’s well-being during times of physical isolation characterized by very limited social contacts outside one’s household. As the COVID-19 related restrictions undermine social resources in general, having such resources within one’s household (e.g., children) is likely to sustain well-being by combating the harmful effects of work-non-work interference. Given the theorizing above, we predict having children to act as a resource and thus buffer the impact of losses in another resource (higher work-non-work interference) on employee well-being:

#### Hypothesis 5

*The association between work-non-work interference and diminished well-being at work (i.e., decreases in work engagement, increases in burnout and job boredom) is weaker for those who have children than for those who do not have children living at home.*

## Method

### Sample and study context

We used two-wave survey data of matched respondents (*N* = 996). At Time 1, before the COVID-19 outbreak in December 2019 and January 2020, a randomized population sample from the Finnish working-age (18–65 years old) population was collected. Altogether 1567 individuals responded at Time 1. In June 2020 (Time 2), approximately three months after the COVID-19 outbreak, 1076 (68.6%) responded to the follow-up survey. We excluded those who were not employed at both time points (*n* = 70) and those who did not report whether their teleworking time had increased (*n* = 10). Analyses did not indicate substantial non-random sampling due to participant dropout (see Supplemental Material A).

Most (67.2%, *n* = 669) reported that they did not have children who live at home at Time 1, whereas *n* = 327 (32.8%) reported that they had one or more children living at home. Amongst those who had children living at home, 41.9% had one child, 40.7% had two, 13.8% had three children, and 3.6% had four to seven children. Most had only children who were school age (seven years old or older; 45.2%), 30.9% had only children who were under school age, and 23.9% had children from both age groups. Amongst those who teleworked at Time 2 (46.9% of the sample), a clear majority (71.9%) reported they teleworked all their working time, 13.9% teleworked three-quarters, 11.5% half, and 2.7% one-quarter of their working time. Amongst those who teleworked at least three-quarters of their working time at Time 2, a majority (89.5%) indicated that they had experienced increases in teleworking since the COVID-19 outbreak in March 2020. Teleworking time was not measured at Time 1 (see Discussion﻿).

On average, participants were 46.1 years old (SD = 10.68), worked 37.4 h a week (SD = 6.82), and had a tenure of 12.2 years (SD = 10.78). Most were women (58.7%), worked in the private sector (53.6%), and had a degree from a university or university of applied sciences (51.9%) whereas 44.1% had upper secondary school or vocational education. Participants were from a range of industries, with the largest ones being the municipal sector (23.3% of respondents), industry and manufacturing (13.2%), government (9.5%), and business services (6.7%). This study was approved by the Ethical Review Committee of the authors’ institution.

Altogether 26.3% of the final sample was collected via postal survey and 73.7% via electronic survey. At Time 1, the postal survey was posted to 2609 individuals who were randomly chosen from the registry of Finnish residents. The response rate for the postal survey was 19.8%. The electronic survey was sent to 6366 individuals who were part of a large existing group of 30 000 online panelists of Finnish residents. The response rate for online panelists was 17.8%. Taloustutkimus Inc., which is an independent market research company operating in Finland, collected the data whereas the authors designed the study. Including the response method (postal/electronic) as a control variable in the hypothesized models did not alter the main findings or the conclusions of this study.

In March 2020, the first wave of COVID-19 hit Finland. The number of detected COVID-19 cases rose steeply from mid-March 2020. This led to notable changes for millions of employees and citizens. On 17 March, The Finnish Government declared emergency powers legislation by which social gatherings of 10 people or more became illegal. Public services such as schools were closed and there was a strong recommendation by the government and health officials to keep also younger children at home, which was widely complied with thus restricting access to daycare. Due to social restrictions and enforced closures of offices, 60.5% of Finnish employees switched to telework from home during Spring 2020 (Eurofound, 2020). In practice, teleworking during COVID-19 took place in employees’ homes (Eurofound, 2020), which was further confirmed by Finnish population surveys showing that 95% of teleworkers reported working from home during this time (Hyry, 2020). Importantly, the reason for the increase in telework during COVID-19 were policies put in place by the employers who followed the strong recommendations from the government and health official to order employees to telework from home wherever possible and thus mitigate the spread of COVID-19 by avoiding physical contact (Milasi et al., 2020). Given this context, the examined increase in telework in this study represents increases in mandatory telework from home which is forced by the circumstances, rather than a work arrangement that is self-selected by the employees in terms of location and timing (Ruohomäki, 2020). The combination of telework and having children at home meant that a vast number of employees had to care for and homeschool their children while simultaneously managing work duties during Spring 2020.

### Measures

We present composite reliability scores, which all were 0.70 and above, and correlations in Tables 1 and 2. We measured the increase in teleworking and the mediational mechanisms at Time 2 with the following instruction preceding the scale items: “Please report changes in your work and non-work life that were brought upon by COVID-19”. By this, we were able to obtain data regarding the impact of COVID-19 after the outbreak (i.e., at Time 2) and dynamism in these experiences and thus have measures compatible with each other as we similarly examined changes in well-being COVID-19 (Ajzen, 1991). To minimize respondent fatigue and retain participation in the study, we used two-item measurements for mediator mechanisms. The items were assessed with a five-point scale (1 = *completely disagree;* 5 = *completely agree*). *Increase in job control* was measured with items “I have made decisions regarding my job more autonomously” and “I have learned new and better working methods” adapted from Karasek et al. (1998) reflecting decision authority and skill discretion, respectively. *Loss of social support* was measured with items “I have received less support from my co-workers” and “I have received less support from my supervisor” which were self-developed. *Work-non-work interference* was measured with items drawn from Fisher et al. (2016) which were rated as having high content validity and research utility; “My work life has frequently interfered with my personal and/or family life” and “My personal and/or family life has frequently interfered with my work life”^2^. *Increase in telework* was measured at Time 2 with an item “The time I spend teleworking has increased since the COVID-19 outbreak”. Altogether 48. % (*n* = 482) of the respondents agreed with this statement.

**Table 1:** Means, standard deviations, composite reliabilities, and zero-order correlations for full sample (*n* = 996)

Variable	Scale	*M*	*SD*	1	2	3	4	5	6	7	8	9	10	11
1. Increase in teleworking	1 − 5	2.88	1.84	−										
2. Increase in job control (T2)	1 − 5	2.95	0.93	0.41***	0.70									
3. Loss of social support (T2)	1 − 5	2.61	1.02	0.26***	0.28***	0.73								
4. Work-non-work interference (T2)	1 − 5	2.05	0.97	0.24***	0.15***	0.36***	0.73							
5. Work engagement (T1)	0 − 6	4.32	1.48	0.04	0.18***	−0.04	−0.14***	0.90						
6. Work engagement (T2)	0 − 6	4.41	1.39	0.06	0.20***	−0.15***	−0.21***	0.76***	0.88					
7. Burnout (T1)	1 − 5	2.15	0.62	−0.04	−0.09**	0.18***	0.33***	−0.62***	−0.55***	0.86				
8. Burnout (T2)	1–5	2.15	0.61	−0.01	−0.12***	0.24***	0.37***	−0.54***	−0.62***	0.80***	0.84			
9. Job boredom (T1)	0–6	2.78	1.49	−0.02	−0.09**	0.16***	0.14***	−0.39***	−0.39***	0.54***	0.51***	0.79		
10. Job boredom (T2)	0–6	2.86	1.47	0.03	−0.13***	0.20***	0.19***	−0.38***	−0.44***	0.47***	0.56***	0.68***	0.82	
11. Having children who live at home^a^	0/1	0.33	0.47	0.14***	0.04	0.02	0.22***	0.04	0.05	−0.05	−0.04	−0.07	−0.07	−

*Note. N* = 996. T1 = Time 1; T2 = Time 2. Composite reliability coefficients are presented on the diagonal. ^a^Having children who live at home was coded as 0 = did not have children who live at home, 1 = had children who live at home.

* *p* < .05

** *p* < .01

*** *p* < .001

**Table 2:** Means, standard deviations, composite reliabilities, and zero-order correlations for those who did not have children who live at home (*n* = 669) and those who did have children living at home (*n* = 327)

	Scale	Did not have children living at home			Had children living at home			Correlations							
Variable		*M*	*SD*	*CR*	*M*	*SD*	*CR*	1	2	3	4	5	6	7	8
1. Increase in teleworking	1 − 5	2.73	1.84	−	3.19	1.82	−	−	0.30***	0.08	0.20***	−0.11*	−0.13**	−0.03	−0.05
2. Work-non-work interference (T2)	1 − 5	1.88	0.89	0.80	2.39	1.03	0.84	0.17***	−	−0.02	−0.02	0.26***	0.28***	0.18***	0.16***
3. Work engagement (T1)	0 − 6	4.27	1.51	0.89	4.43	1.43	0.90	0.01	−0.22***	−	0.68***	−0.53***	−0.48***	−0.34***	−0.35***
4. Work engagement (T2)	0 − 6	4.35	1.44	0.89	4.54	1.26	0.85	−0.01	−0.31***	0.78***	−	−0.46***	−0.55***	−0.37***	−0.35***
5. Burnout (T1)	1 − 5	2.16	0.63	0.86	2.11	0.59	0.86	0.00	0.39***	−0.66***	−0.57***	−	0.77***	0.54***	0.47***
6. Burnout (T2)	1–5	2.17	0.62	0.86	2.11	0.58	0.79	0.05	0.44***	−0.56***	−0.65***	0.81***	−	0.49***	0.52***
7. Job boredom (T1)	0–6	2.82	1.51	0.79	2.71	1.43	0.77	−0.01	0.15***	−0.41***	−0.39***	0.54***	0.51***	−	0.68***
8. Job boredom (T2)	0–6	2.92	1.50	0.85	2.74	1.40	0.74	0.08*	0.23***	−0.39***	−0.47***	0.46***	0.58***	0.68***	−

*Note.* Correlations for those who did not have children living at home (*n* = 669), are below the diagonal, and for those who did have children living at home (*n* = 327) are above the diagonal. T1 = Time 1; T2 = Time 2. CR = Composite reliability coefficient.

* *p* < .05

** *p* < .01

*** *p* < .001

The multiple types of employee well-being were assessed at Time 1 and Time 2. We measured *work engagement* with a short version of the Utrecht Work Engagement Scale by Schaufeli et al. (2017) comprising three items (e.g., “At my work, I feel bursting with energy”) that tapped into experiences of vigor, dedication, and absorption at work. *Job boredom* was measured with three items adapted from the Dutch Boredom Scale by Reijseger et al. (2013). The items were “During work time I daydream”; “At work, time goes by very slowly”; and “I feel bored at my job” which represented the three aspects of job boredom; behavioral, cognitive, and affective, respectively. Both work engagement and job boredom were assessed on a seven-point scale (0 = *never*; 6 = *daily*). We measured burnout with the Burnout Assessment Tool (Schaufeli et al., 2020) comprising 23 items, which reflected exhaustion (eight items; e.g., “At work, I feel mentally exhausted”), mental distance (five items, e.g., “I struggle to find any enthusiasm for my work”), cognitive impairment (five items, e.g., “At work, I struggle to think clearly”) and loss of emotional control (five items, e.g., “At work, I feel unable to control my emotions”). Burnout items were assessed on a five-point scale (1 = *completely disagree;* 5 = *completely agree*).

### Statistical analysis

We tested our hypotheses by Latent Change Score (LCS) modeling (McArdle, 2009) in Mplus 8.4 (Muthén & Muthén, 2017). In these structural equation models, we used maximum-likelihood robust estimation with robust standard errors as it is robust to non-normality and estimated covariances between residuals of the same items across time. Model comparison analyses were tested by the Satorra-Bentler chi-square difference test (Satorra & Bentler, 2001). LCS was the most suitable analytical method as it enabled us to model within-person changes across two time points in the outcome constructs and to test the hypothesized mediational mechanisms and differences between groups by multigroup modeling. LCS factors were construed by (a) regressing the latent Time 1 score on Time 1-Time 2 latent change score, (b) regressing Time 1 score on Time 2 score with a fixed estimate of 1, and (c) regressing the Time 1-Time 2 latent change score on Time 2 score with a fixed estimate of 1 and setting the residual of Time 2 to zero (for more detailed description, see McArdle, 2009). Importantly, LCS models do not suffer from the same methodological limitations as residual change scores or difference scores (Henk & Castro-Schilo, 2016). We used weighting in terms of age, gender, and residential area in the analyses to match the population distribution.

As the hypotheses concern associations both in the full sample (H1-H3) and amongst two groups (H4, H5), testing such hypotheses necessitates separate statistical models. In Model 1, we test Hypotheses 1–3. In Model 2, a multigroup model, we test Hypotheses 4 and 5, which predict differences between those who have and did not have children living at home. Differences between groups were tested by using the model constraint command in Mplus in which the path estimate of the group ‘do not have children’ was subtracted from the path estimate of the group ‘have children’. For hypothesis testing for job control, social support, and work-non-work interference we used composite scores as single indicators of latent variables to avoid technical issues when modeling latent variables with two indicators (see Brown, 2015). To account for the measurement error also in these three single indicator latent variables, we set the indicator residuals to 1–composite reliability coefficient as recommended by Kline (2016). To evaluate good model fit, we followed the general guidelines of acceptable values of Comparative Fit Index (CFI) and Tucker-Lewis Fit Index (TLI) above 0.90 and Root Mean Square Error of Approximation (RMSEA) and Standardized Root Mean Square Residual (SRMR below) 0.10 (e.g., Brown, 2015; Kline, 2016).

## Results

### Preliminary analyses

Confirmatory factor analyses supported the hypothesized factor model and measurement invariance tests supported partial strict measurement invariance over time and between groups (see Supplemental Material B). These findings suggested that the scale items were not interpreted differently at different time points or between those who had or did not have children.

### Hypotheses tests

**Fig. 1:**
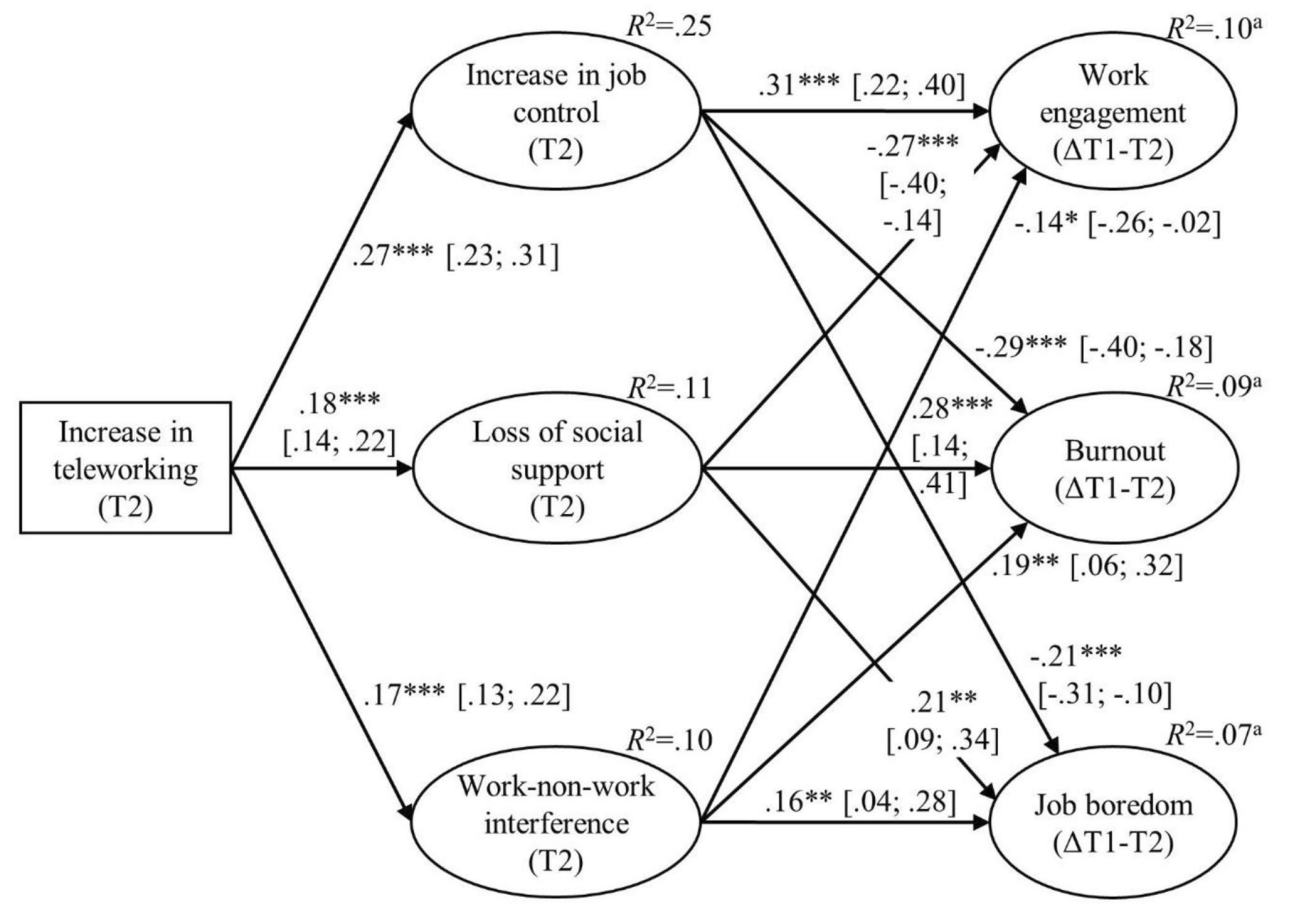
Full mediation latent change score model (Model 1). *N* = 996. Standardized path estimates with 95% confidence intervals in brackets are presented. T1 = Time 1; T2 = Time 2. Symbol Δ refers to within-person changes. For clarity, omitted from the figure are, latent factors’ items, latent factors at Time 1 and Time 2 of the three latent change scores, and residual covariances between the three mediator latent factors and between the three outcome latent change scores. ^a^The *R*^2^-value for the three latent change scores represents the amount of variance explained by the three predictors (increase in job control, loss of social support, work-non-work interference) as the presented *R*^2^-value excludes the variance explained by the Time 1 score of the latent change score (e.g., burnout Time 1), which is regressed on the latent change score (e.g., burnout ΔT1-T2) to estimate within-person changes in the outcome construct (e.g., burnout) * *p* < .05; ** *p* < .01; *** *p* < .001

First, we tested hypotheses 1–3 in Model 1. The full mediation model (Fig. 1) provided an acceptable fit with the data, χ²(233) = 824.08, *p* < .001, CFI = 0.93, TLI = 0.92, RMSEA = 0.05, SRMR = 0.06. Hypotheses regarding the indirect effects from increases in teleworking to changes in well-being at work via increase in job control (Hypothesis 1), loss of social support (Hypothesis 2), and work-non-work interference (Hypothesis 3) received support as the confidence intervals did not include zero and the direction of the effects was as hypothesized (Table 3).

**Table 3:** Standardized coefficients for indirect effects

Indirect Path	Standardized coefficient	95% Confidence Interval
Increase in teleworking → Increase in job control → ΔWork engagement	0.08	[0.06; 0.11]
Increase in teleworking → Increase in job control → ΔBurnout	−0.08	[− 0.11; −0.05]
Increase in teleworking → Increase in job control → ΔJob boredom	−0.06	[− 0.08; −0.03]
Increase in teleworking → Loss of social support → ΔWork engagement	−0.05	[− 0.08; −0.03]
Increase in teleworking → Loss of social support → ΔBurnout	0.05	[0.03; 0.09]
Increase in teleworking → Loss of social support → ΔJob boredom	0.04	[0.01; 0.07]
Increase in teleworking → Work-non-work interference → ΔWork engagement	−0.02	[− 0.05; −0.01]
Increase in teleworking → Work-non-work interference → ΔBurnout	0.03	[0.01; 0.06]
Increase in teleworking → Work-non-work interference → ΔJob boredom	0.03	[0.01; 0.06]

Note. The number of samples = 10 000. Bias-corrected 95% confidence interval.

**Fig. 2:**
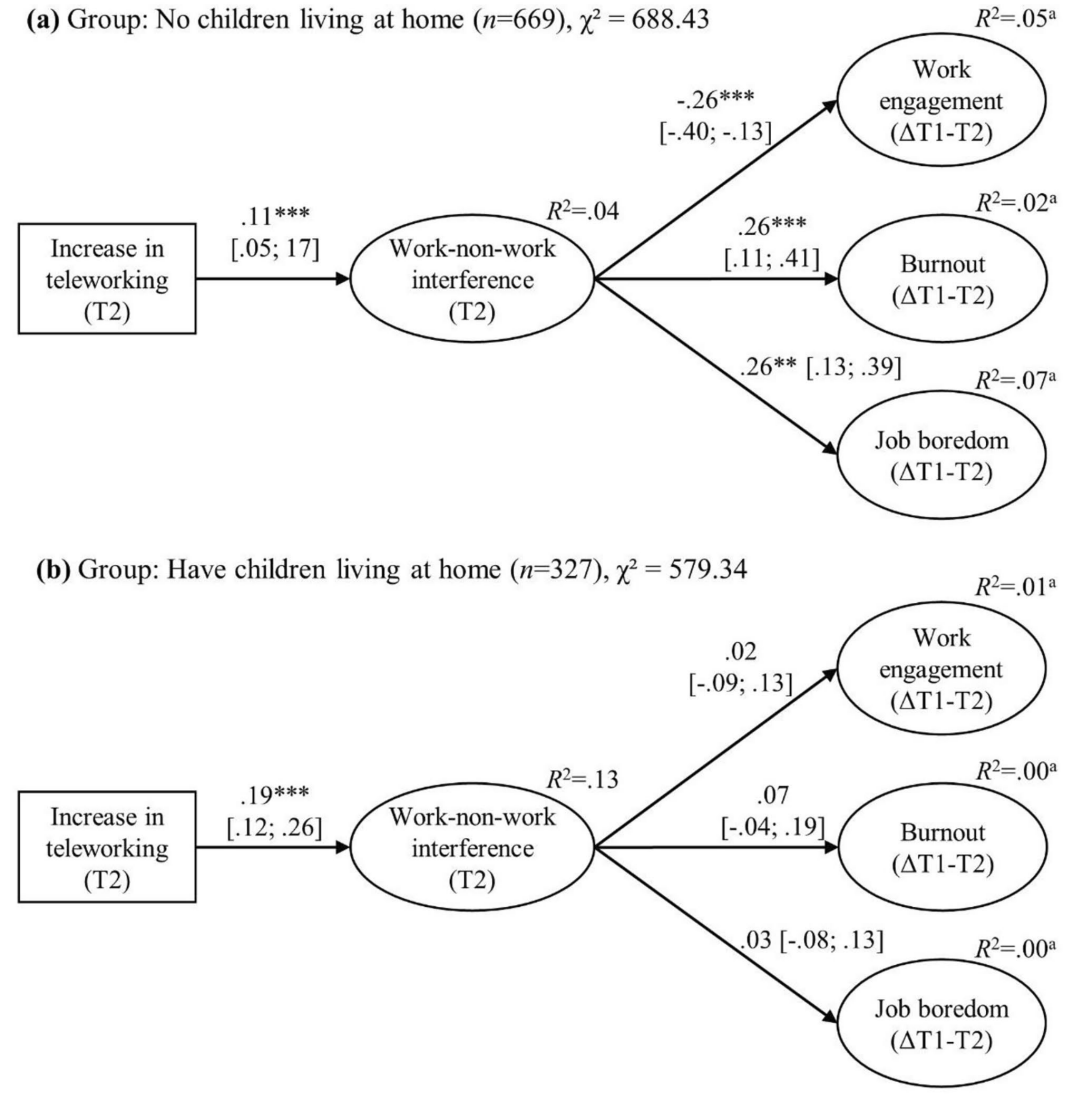
Multigroup latent change score model (Model 2). Standardized path estimates with 95% confidence intervals in brackets are presented. T1 = Time 1; T2 = Time 2. Symbol Δ refers to within − person changes. For clarity, omitted from the figure are latent factors’ items, latent factors at Time 1 and Time 2 of the three latent change scores and residual covariances between the three outcome latent change scores ^a^The *R*^2^-value for the three latent change scores represents the amount of variance explained by work-non-work interference as the presented *R*^2^-value excludes the variance explained by the Time 1 score of the latent change score (e.g., burnout Time 1), which is regressed on the latent change score (e.g., burnout ΔT1-T2) to estimate within-person changes in the outcome construct (e.g., burnout) * *p* < .05; ** *p* < .01; *** *p* < .001

The multigroup latent change score model is presented in Fig. 2. The model provided an acceptable fit with the data, χ²(445) = 1267.02, *p* < .001, CFI = 0.91, TLI = 0.91, RMSEA = 0.06, SRMR = 0.08. The path estimate from increases in teleworking and work-non-work interference was statistically significantly higher (see Fig. 2) for those who had children living at home in comparison to those who did not have children, diff = 0.10, 95% CI [0.02; 0.18]. Thus, Hypothesis 4 was supported. The path estimates from work-non-work interference to changes in well-being at work were also all statistically significantly different between the groups, diff = − 0.21, 95% CI [− 0.34; −0.08] for work engagement, diff = 0.14, 95% CI [0.01; 0.28] for burnout, and diff = 0.23, 95% CI [0.08; 0.39] for job boredom. Given the path estimates shown in Fig. 2, Hypothesis 5 was supported as the associations from work-non-work interference to changes in work engagement, burnout, and job boredom were weaker for those who had children in comparison to those who did not have children.

### Post-hoc analyses

We examined whether various demographic variables played a role in our research model. Studies have suggested that occupational well-being during COVID-19 and work-non-work interference could vary as a function of gender (Shockley et al., 2020; Zacher & Rudolph, 2021). Furthermore, younger employees may have suffered the most from the COVID-19 driven social restrictions (Evans et al., 2021). We therefore re-analyzed the statistical models by including age and gender as control variables by regressing them on the three mediator and outcome variables. These analyses did not alter the main conclusions of the study and none of the paths estimated from age and gender were statistically significant. Second, amongst those who had children, we examined the number of children, ages of children (whether at school-age or younger), and gender. Adding these variables as predictors did not change the main findings and none of these paths were statistically significant. Please contact the first author for detailed results.

## Discussion

By using a repeated-measures quasi-experimental design in a randomized population sample and analyses of within-person changes in employee well-being before and after the COVID-19 outbreak, we capture the dynamics that unfolded in the early stages of the pandemic. By this, we provide new theoretical and empirical insights regarding the challenges and opportunities in fostering teleworkers’ well-being when employees themselves cannot decide whether to telework or not. In addition to informing the application of theories in the telework literature, our findings are relevant for practice as mandatory telework and hybrid work arrangements as work practices are likely considered and applied by numerous organizations also after the immediate effects of the pandemic. For instance, employees may be ‘forced’ to telework also due to closures or lack of office spaces or long commuting distances.

All hypotheses received support. The increase in teleworking was associated with improved well-being in terms of increases in work engagement and decreases in burnout and job boredom via more increase in job control. Furthermore, the association between increases in teleworking and the deterioration of employee well-being was mediated by a greater loss of social support at work and higher work-non-work interference. Although having children who live at home appeared to be harmful to employees by accentuating the association between the increase in teleworking and work-non-work interference, having children also buffered the association between work-non-work interference and decreases in well-being at work.

### Mandatory teleworking may be more likely to disrupt social relationships at work and introduce work-non-work interference

Aligning with the central tenet of COR theory of individuals striving to attain beneficial resources (Hobfoll, 1989), self-selected telework is arguably more likely to produce resources. This is because when teleworking out of volition, employees can arrange their work according to their preferences. Indeed, findings from the pre-COVID-19 era suggest that teleworking generally has positive effects (e.g., Allen et al., 2015; Vega et al., 2015). However, we illuminate how a non-self-selected increase in telework represents an ambiguous and environment-changing event and is thus more prone to lead to losses in the same resources and thus decrease employee well-being (Hobfoll, 1989). Our findings corroborate other results from studies suggesting that teleworking during the COVID-19 outbreak has more negative effects on employees than teleworking before the outbreak (Kaluza & van Dick, 2022). These findings challenge the current understanding of teleworking and suggest that for theorizing and studying the effects of telework, it is important to note that “theoretical meanings and relationships may have been shaped or changed by the unique context” (Wang et al., 2021, p. 45). Wang et al. (2021) further elaborate: “…we advocate that it is theoretically and practically important to regard remote working during the pandemic as a context and explore/examine what virtual work characteristics really matter and how they matter” (p. 45), which are questions we address in the current study.

While in their meta-analysis Gajendran and Harrison (2007) found that teleworking was positively associated with the employee-supervisor relationship, our results suggest that increases in teleworking were associated with *loss of* social support from supervisors and colleagues. This finding suggests that when teleworking is not similarly self-selected and flexible as before COVID-19, teleworking more likely leads to loss of social resources thus effectively preventing employees to attain or maintain such resources. However, as nearly all the 46 studies included in the meta-analysis were cross-sectional, the found association by Gajendran and Harrison (2007) may also be due to supervisors allowing teleworking for employees with whom they already have high-quality relationships, rather than teleworking having a positive effect on relationships. In our study, the reason for teleworking was COVID-19 related restrictions rather than decisions made by the supervisors. Furthermore, we asked respondents whether they experienced decreases in social support since the COVID-19 pandemic. For these reasons, our study may provide more robust evidence regarding the impact of teleworking on social support as a resource loss. This notion is further emphasized by the fact that the current knowledge on teleworking is overwhelmingly based on cross-sectional studies (Charalampous et al., 2019; Oakman et al., 2020) which provide only very limited information regarding cause-and-effects and the extent and reasons why teleworking impacts well-being over time.

Our finding of teleworking being associated with higher work-non-work interference is also in contrast to the majority of findings before COVID-19, which have shown that on average there is a relatively small association between telework and *lower* work-non-work interference (Allen et al., 2013). Prior research has accordingly suggested that teleworking from home could be a tool for maintaining work-life balance (e.g., Hill et al., 2003). When employees are in control of the location and scheduling of telework, it is more likely that teleworking helps with balancing the work and non-work domains of life by introducing flexibility in role boundaries (Gajendran & Harrison, 2007). Our findings show that this may not be the case during forced telework from home as it appears to harm the attainment of such resources (e.g., work-life balance) rather than provide a way to gain such beneficial resources. By this, our study shows how mandatory telework from home may harm well-being via higher work-non-work interference and thus also address calls to examine the long-term mental health outcomes of work-non-work interference (Allen & Martin, 2017). Our results further corroborate the findings by Palumbo (2020) and Sandoval-Reyes et al. (2021) who found a negative cross-sectional association between working from home during COVID-19 and work-life balance.

In contrast to social support and work-non-work interference, our finding of increase in job control amongst teleworkers aligns with existent literature (e.g., Charalampous et al., 2019). This speaks for the notion that gain in autonomy and learning seemingly does not depend on whether teleworking is self-selected or forced. Our findings also suggest that increased job control was the strongest mediational mechanism given the highest indirect effects (Table 3), which also differed statistically significantly from other indirect paths at *p* < .001 (please contact the first author for detailed results). This indicates that increases in telework during COVID-19 may to some extent more likely benefit well-being via fostering job control than harm well-being via loss of social support and higher work-non-work interference.

### Family both as “an ally and as an enemy” when teleworking during COVID-19

By drawing from two competing frameworks, that is, role depletion (Greenhaus & Beutell, 1985) and role enrichment (e.g., Marks, 1977) theories, we expected having children to play a dual role in terms of employees’ well-being. Whereas increases in teleworking were associated with higher work-non-work interference for those who have and those who do not have children living at home, this association was particularly evident amongst those who had children at home. Existent findings have shown that having children or other people in the same household when working from home is associated with higher work-non-work interference (Allen et al., 2021; Byron, 2005; Michel et al., 2011). To the best of our knowledge, this is the yet first study to suggest that having children accentuates the impact of telework on work-non-work interference. This lends support to the proposition put forth by role depletion theory.

However, and importantly, having children at home appeared to also buffer the negative impact of higher work-non-work interference on all the examined types of employee well-being as we hypothesized based on the role enrichment theory. To our knowledge, this is a unique finding which illuminates in a new way the potential beneficial role that having children may have for employees’ well-being. However, Blahopoulou et al. (2022) found that having under 18 years old children was associated with better well-being among teleworkers during COVID-19. Albeit the authors examined a direct association rather than a moderating effect as in the current study, interestingly their findings point to the same direction: having children benefits well-being. Employees with children may have enjoyed the increased time spent with their children, which may be associated with several positive aspects of life such as greater involvement in family activities and improved emotional connections (Rudolph et al., 2021). However, we note that the context of our study may have accentuated the found moderation effects of having children at home. Speculatively, during times when there are no restrictions for schools and daycare, having children may not similarly accentuate the impact of telework on work-non-work interference. Also, when it is possible to have contacts also outside one’s household, the role of having other people within the household for well-being may not be so significant.

As a theoretical insight into COR theory (Hobfoll, 1989), we drew from role enrichment and depletion theories to argue that having children may act both as a beneficial resource and as a demanding environmental factor that may threaten other resources. Our results emphasize the argument that notions from COR theory must be viewed in a specific context and that integrating other theoretical frameworks are valuable for identifying how specific conditions, such as having children, may either act as a resource or have even opposite effects (Halbesleben et al., 2014; Hobfoll et al., 2018). Regarding role depletion and enrichment theories, our results suggest that it is not that one theory is correct and the other is wrong. Rather, in one instance having children may accentuate negative impacts by depleting energies, whereas in another instance having children may buffer such negative impacts by enriching one’s life. This study therefore draws bridges between these two frameworks and thus expands our current theoretical understanding of the topic. Researchers are advised to consider the benefits of synthetization of these seemingly contradictory frameworks as it likely provides novel insights into the work-non-work literature.

### Engaged, burned out, or bored out due to telework?

Our results suggest that increases in teleworking may lead to increases or decreases in various types of employee well-being dimensions, both positive and negative states and that by ensuring job control, social support at work, and work-life balance, it is possible to simultaneously reinforce employees’ work engagement and mitigate burnout and job boredom. By this, we provide a fuller understanding regarding the impact that telework may have on occupational well-being as the majority of teleworking studies have examined only one type of well-being in a given study (e.g., strain or job satisfaction; Charalampous et al., 2019).

To the best of our knowledge, this is the first study to examine associations between telework and job boredom. Even though job boredom is a scarcely studied state of well-being, it is essential to understand the causes of boredom as it bears many negative outcomes for the individual (e.g., poor health) and organization (e.g., higher turnover intentions and lower organizational commitment; Harju et al., 2014; Reijseger et al., 2013). Focusing solely on how to foster work engagement and prevent burnout at work, likely overshadows other states of ill-being and thus hinders the development of a more holistic understanding of well-being at work.

### Practical implications

Our findings provide essential insights for employers and employees alike in the context of telework which is not self-selected by the employees. Even without a global pandemic forcing employees to telework from their homes, mandatory telework may occur also after the pandemic for instance due to a lack of office space available for the employees.

It is important to consider that mandatory teleworking may both benefit and harm various facets of employee well-being. By ensuring job control, that is autonomous decision-making and learning new ways of working, the impact is more likely beneficial. Job control is facilitated by leadership practices that provide employees with a sense of power and foster proactivity and self-confidence (van Dierendonck, 2011) and delegation of responsibilities (Stoker et al., 2021). Conversely, employees’ autonomy and discretion may be harmed if the employer uses remote surveillance methods.

When teleworking is mandatory, it may be especially important to pay attention to employees’ social resources and relationships at work and balance between work and non-work domains of life, which are likely to hinder employees’ well-being and motivation at work. To facilitate social support, organizations are advised to build a culture that emphasizes caring and relationships (Groysberg et al., 2018). Employees can also proactively craft more support by asking for advice and help (Tims et al., 2013) or by showing consideration to others and by improving collaboration at work (Kaltiainen et al., 2022). Employers may increase employees’ work-non-work balance through flexible work hours, managerial support, and family-friendly policies (Breaugh & Frye, 2008; Kossek et al., 2011; e.g., Rofcanin et al., 2020) and by taking into account employees’ integration preferences (Palm et al., 2020). Employees may apply “rites of passage”, which may be physical (e.g., taking a walk before and after the workday) or psychological (e.g., noting the accomplished work tasks after the workday), that can help in transitioning from one role to another (Ashforth et al., 2000) and coping strategies including concentrating their efforts, asking for support, and trying various ways to achieve their goals (Baltes et al., 2011).

Our findings also suggest that employers and societies are advised to pay special attention to both groups, those who have and do not have children living at home, but for different reasons. Those who do not have children, and potentially also those who live alone, may more likely experience negative well-being consequences because of an imbalance between work and non-work domains of life. This may be especially so when contacts with people outside one’s household are restricted. Potentially, keeping constant touch with such employees and providing them experiences of belongingness with other people or with the work organization may be important. At the same time, our findings suggest that when teleworking is not self-selected by the employees, organizations are advised to provide additional support for employees with children (e.g., provide childcare services, flexible working time) as they are more at risk of experiencing higher work-non-work interference due to telework increase. This is important as work-non-work interference may have negative consequences beyond the examined employee well-being dimensions (e.g., lower performance and career satisfaction and success, higher turnover; Eby et al., 2005; Vaziri et al., 2020).

### Limitations and future research

Despite the strengths of our study, our study is not without limitations. Even though we took the baseline levels of the main outcome variables into account as we modeled changes in them over time and for this part of our model used a repeated-measurement design with established strict measurement invariance, our findings are correlational and cannot establish causality. Causal inferences are further limited as we cannot be certain that we have not omitted a variable that could explain some of the found associations. Another potential methodological limitation is common method bias, which is an inherent part of research using a single-source method such as our study. However, our use of repeated measures across different circumstances and emphasis on confidentiality to the participants may have mitigated this risk to some extent (Spector, 2006). We note that obtaining information about employees’ motivational and affective states and perceptions of work and non-work characteristics necessitates the use of self-report.

Furthermore, capturing the three mediational mechanisms with more items could have increased the nuance and accuracy of our findings further. While measures with more items are psychometrically preferable, fewer items may capture the examined construct sufficiently and at the same time reduce respondent attrition (Fisher et al., 2016). The psychometric properties of these scales were all acceptable as all composite reliability scores were 0.70 and above (see Table 1) and the measurement model provided an acceptable fit with the data (see Supplementary Material B). Also, it would have been ideal to estimate the changes in the mediator mechanisms and teleworking by measuring them at both time points and then statistically estimate the extent of changes, as we did for employee well-being dimensions, rather than rely on retrospective assessments of changes at Time 2. However, we did not measure these variables at the baseline (Time 1) as this research project was not originally designed to examine the impact of changes in teleworking on well-being via changes in work and non-work characteristics as the project was launched in Fall 2019. Retrospective measures of changes may overestimate the extent of such changes (Young et al., 2022). However, we did not draw conclusions regarding the mean levels of these variables or changes, which may be biased, but rather associations between the variables.

We call for future research to examine the interactions and joint effects of the amount and changes in teleworking. Such a study would necessitate a setting in which increases in teleworking would be more evenly distributed amongst teleworkers. Given that during COVID-19 those who were able to telework were in general forced to increasingly do so, the current study cannot address this question. In our sample, there were not many teleworkers who had not experienced increases in teleworking (see Sample and study context) and the variables of increases in teleworking since COVID-19 outbreak and amount of teleworking at Time 2 correlated at *r =* .81, *p* < .001, thus suggesting that for those who were able to telework, teleworking time had increased, which is understandable given the context of our study. Future studies would also benefit the current understanding by examining the familiarity with telework before such an increase. We would expect that the more dramatic the shift to teleworking is, the more it affects the characteristics of work and non-work and as a result employee well-being. A study by Eurofound (2020) found that 14.5% of Finnish employees had teleworked from home daily or several times a week before COVID-19, suggesting that for the majority of our respondents working from home was a relatively new work arrangement. Future studies could also verify our findings by measuring the extent that the participant is able to self-select the amount of teleworking. While our study lacks such measures, teleworking in the context of our study largely occurred due to closures of office spaces and other government-enforced COVID-19 policies restricting social interaction and was therefore not similarly self-selected as in environments before the global pandemic. For instance, a clear majority of the teleworkers in our sample teleworked full time, and other studies have shown that teleworking in Finland during the time span of our study was forced rather than self-selected (see Sample and study context).

Several variables could shed further light on the examined phenomena and would be important to examine in future research. For instance, employees may vary in their boundary management preferences and behaviors (e.g., Reinke & Gerlach, 2021), and especially for those who prefer segmentation roles, the interference between work and non-work domains may be especially harmful (Allen et al., 2014). Yet, a recent study found that the number of people living in one’s household was associated with work-non-work imbalance despite boundary management preferences (Allen et al., 2021). Also, for those with a separate office space at home, increase in teleworking may not similarly increase work-non-work interference.

Future research would also benefit from more fine-grained analysis regarding the ages of children, as younger children typically require more attention and childcare thus potentially leading to higher work-non-work interference amongst employees teleworking from home. However, a meta-analysis by Michel et al. (2011) did not find an association between the age of children and work-family conflict. Potentially single-guardians with children experience more work-non-work interference than households with more caretakers. Furthermore, whereas our study suggests beneficial family dynamics, we did not examine perceptions of work-family enrichment which posits that permeability between these roles may lead to positive spillovers (McNall et al., 2010). Perhaps those who had children experienced more such enrichment, which could illumine further the current findings (Peeters et al., 2005). Also, while employee well-being typically stems from job characteristics, falling severely ill or worrying about the well-being of others may have impacted how employee well-being evolved in the context of our study, during the COVID-19 pandemic.

## Conclusion

This study identified three mechanisms – increase in job control, loss of social support, and higher work-non-work interference – which may explain why increases in teleworking during COVID-19 may both harm and benefit well-being at work. Whereas prior telework literature has often examined telework based on employee preferences, our findings illuminate how telework that is forced upon rather than self-selected may have a more negative impact on social support at work and work-non-work interference and thus pose a greater risk for employee well-being. We also synthesize the role depletion and role enrichment theories, as we show how having children at home can both be a stressor and a resource when teleworking from home. Moreover, work-family research has largely examined subjective perceptions (Peeters et al., 2005), thus neglecting home-related objective structural factors. Our study suggests that at least one such structural factor, having children at home, may play an important and complex role in shaping employee well-being.

## Electronic supplementary material

Below is the link to the electronic supplementary material.

Supplementary Material 1

Supplementary Material 2

## Data Availability

The data is available from the corresponding author on reasonable request.

## Footnotes

1 We use the concept of work-non-work interference as an umbrella term that incorporates both negative impacts from work to non-work and from non-work to work. Whereas the directionality of the interference has been separated conceptually (Allen et al., 2013; Shockley & Allen, 2007), we are not aware of theoretical rationale positing that the impact on employee well-being would depend on the direction. We thus follow existing practices that combine both aspects into the same measure (see Netemeyer et al., 1996).

2 We note that the phrasing of the two items used to measure work-non-work interference were not phrased to explicitly refer to changes in the phenomenon.
