# Supplementary material for: Why increase in telework may have affected employee well-being during the COVID-19 pandemic? The role of work and non-work life domains
Source: Curr Psychol. 2023 Jan 26:1–19. Online ahead of print. doi: 10.1007/s12144-023-04250-8 (PMC9878489; doi:10.1007/s12144-023-04250-8)
Supplement: Supplementary file 1 — Supplementary Material 1 [file 12144_2023_4250_MOESM1_ESM.pdf]

Supplemental Material, Appendix A: Attrition analyses

To assess whether there was non-random attrition in the sample, we examined whether the mean levels of the hypothesized variables measured at Time 1 and demographics differed between those who responded only at Time 1 ($n = 490$) and those who responded at both time points and were included in the final data ($n = 996$). The mean levels of the hypothesized variables did not differ statistically significantly between these two samples as shown by p-values of t-tests for work engagement, burnout, and job boredom, which were $p = .324$, $p = .957$, and $p = .442$, respectively. The two samples were also largely similar in terms of demographics. There were no statistically significant differences in gender ($p = .700$), whether the participant had a permanent or temporary work contract ($p = .298$), and in the amount of weekly working hours ($p = .695$). Those who responded at both time points, were slightly older ($M = 42.5$) than those who responded only at Time 1 ($M = 40.4$), $t(1484) = 3.20$, $p = .001$, had a higher education (53 % of the sample) than those who responded only at Time 1 (43 % of the sample), $t(1479) = 3.72$, $p < .001$, had slightly higher years of tenure ($M = 12.04$) than those who responded only at Time 1 ($M = 11.74$), t(1295) = 2.02, $p = .044$, and more often did not hold a supervisory or management position (85 % of the sample) than those who responded only at Time 1 (79.2 % of the sample), $t(1480) = -2.86$, $p = .007$). Given that the levels of the hypothesized variables did not differ between the samples, and the found differences in the demographics were relatively small, these results did not indicate a substantial non-random sampling due to attrition across time.
