# Supplementary material for: Why increase in telework may have affected employee well-being during the COVID-19 pandemic? The role of work and non-work life domains
Source: Curr Psychol. 2023 Jan 26:1–19. Online ahead of print. doi: 10.1007/s12144-023-04250-8 (PMC9878489; doi:10.1007/s12144-023-04250-8)
Supplement: Supplementary file 2 — Supplementary Material 2 [file 12144_2023_4250_MOESM2_ESM.pdf]

Supplemental material B: Measurement models

In the measurement model, latent variables of job control, social support, and work-non-work interference were created with two observed items (i.e., indicators) each. Work engagement and job boredom latent variables were modeled with three indicators each at both time points. For burnout, the items reflecting each of the four symptoms of burnout (seven items for exhaustion and five items for cynicism, cognitive impairment, and emotional control each) were used to create four composite scores, which were used to model the latent burnout variable at both time points.

Regarding alternative factor models to the hypothesized factor model, a model with items of work engagement, burnout, and job boredom loading on the same factor led to substantially worse model fit, $\Delta\chi^2(28)=1901.63$, $p<.001$. The same was found for models with work engagement and burnout items loading on the same factor and job boredom as a separate factor, $\Delta\chi^2(15)=660.22$, $p<.001$, work engagement and job boredom items loading on the same factor and burnout as a separate factor, $\Delta\chi^2(15) =802.98$, $p<$ 001, and burnout and job boredom items loadings on the same factor and work engagement as a separate factor, $\Delta\chi^2(15)=376.83$, $p<.001$.

Supplemental Table 1. Measurement invariance test over time for the full sample.

Model	χ^2	*df*	CFI	TLI	RMSEA	SRMR	$\Delta\chi^2$	*p*
Configural[a]	918.92***	249	.93	.91	.05	.06		
Weak invariance over time[b]	931.37***	256	.93	.91	.05	.06	12.16	.095
Strong invariance over time[c]	965.08***	263	.92	.91	.05	.06	35.16	< .001
Partially strong invariance over time[d]	934.44***	261	.93	.91	.05	.06	1.38	.926
Strict invariance over time[e]	955.68***	274	.92	.91	.05	.06	29.89	.005
Partially strict invariance over time[e]	946.88***	273	.93	.91	.05	.06	26.10	.073

Note. N = 996. [a]A model without constraints. [b]A model with equal item loadings over time. [c]A model with equal item loadings and intercepts over time. [d]A model with equal item loadings and intercepts over time except for intercepts of “At my work, I feel bursting with energy” of work engagement and exhaustion score of burnout scale estimated freely over time. [d]A model with equal item loadings, intercepts, and residuals over time. [e]A model with equal item loadings, intercepts, and residuals over time except for residuals of “At my work, I feel bursting with energy” of work engagement scale. Model comparisons were carried in a stepwise manner, so that at each step a more restricted model was compared with a previous, less restricted model. *** $p < .001$

[a]Based on the model modification indices, in the configural model we estimated covariances among the residuals of the following four item pairs:

- Residuals of scores of emotional impairment and cognitive impairment of burnout scale at Time 1 and at Time 2
- Residuals of scores of exhaustion and cynicism of burnout scale at Time 1
- Residuals of items “During work time I daydream” and “I feel bored at my job” of job boredom scale at Time 2

Supplemental Table 2. Measurement invariance test over time and for the two groups of having and not having children who live at home.

Model	χ^2	*df*	CFI	TLI	RMSEA	SRMR	$\Delta\chi^2$	*p*
Configural[a]	1151.18***	350	.92	.89	.07	.07		
Weak invariance between groups[b]	1159.83***	365	.92	.90	.07	.07	16.77	.333
Weak invariance between groups and over time[c]	1173.22***	372	.92	.90	.07	.07	13.92	.053
Strong invariance between groups[d]	1202.52***	387	.92	.90	.07	.07	29.77	.013
Partially strong invariance between groups[e]	1183.03***	386	.92	.90	.06	.07	11.68	.632
Partially strong invariance between groups and strong invariance over time[f]	1224.66***	396	.91	.90	.07	.07	42.63	< .001
Partially strong invariance between groups and over time[g]	1186.73***	394	.92	.90	.06	.07	4.23	.836
Strict invariance between groups[h]	1197.68***	416	.92	.91	.06	.07	31.83	.080
Strict invariance between groups and over time[i]	1210.86***	426	.92	.91	.06	.07	19.14	.039
Strict invariance between groups and partially strict invariance over time[j]	1199.49***	425	.92	.91	.06	.07	10.33	.324

Note. Do not have children living at home n = 669, have children living at home n=327. [a]A model without constraints. [b]A model with equal item loadings between groups. [c]A model with equal item loadings between groups and over time. [d]A model with equal item loadings between groups and over time and intercepts between groups. [e]A model with equal item loadings between groups and over time and equal intercepts between groups except for the item intercept of "My work life has frequently interfered with my personal and/or family life" of work-non-work interference was estimated freely between groups. [f]A model with equal item loadings and intercepts between groups and over time. [g]A model with equal item loadings and intercepts between groups and over time except for the item intercepts of "At my work, I feel bursting with energy" of work engagement scale and exhaustion score of burnout scale were estimated freely over time. [h]A model with equal item loadings and intercepts between groups and over time and equal residuals between groups. [i]A model with equal item loadings, intercepts, and residuals between groups and over time. [j]A model with equal item loadings, intercepts, and residuals between groups and over time except for the item residuals of "At my work, I feel bursting with energy" was estimated freely over time. Model comparisons were carried in a stepwise manner, so that at each step a more restricted model was compared with a previous, less restricted model. *** $p < .001$

[a]Based on the model modification indices, in the configural model we estimated covariances among the residuals of the following six item pairs:

- Residuals of scores of emotional impairment and cognitive impairment at Time 1, and cynicism and exhaustion at Time 1 for the group of do not have children living at home.
- Residuals of scores of emotional impairment and cognitive impairment at Time 2, and cynicism and exhaustion at Time 1 for the group have children living at home.
- Residuals of items "During work time I daydream" and "I feel bored at my job" of job boredom scale at Time 2 for the group of not having children living at home.
- Residuals of items ""At work, time goes by very slowly" and "I feel bored at my job" of job boredom scale at Time 2 for the group of having children living at home.

The confirmatory factor analysis revealed that some of the item residuals of the measured employee well-being dimensions covaried (see table notes of Supplemental Tables 1 and 2). For instance, the residuals of the items “During work time I daydream” and “I feel bored at my job” of the job boredom scale covaried. We see that the found residual covariances may suggest that some of the items share similar content (Byrne, 2012). Furthermore, items that are similarly worded and measured with a common method, such as a self-report survey as in this study, may result in a “method effect” (Brown, 2015) which in this case is another plausible reason for the found residual covariances. Following recommendations by Brown (2015) and Byrne (2012), we decided to estimate those residual covariances which were supported by substantive and empirical rationale and proceeded with the re-specified factor model to test our hypotheses (see also Bentler & Chou, 1987; Cole et al., 2007). Importantly, we re-analyzed the hypothesized models without any modifications to the model, that is, excluding the residual covariances and setting all the loadings, intercepts, and residuals equal across time and between groups (i.e., setting strict measurement invariance rather than partially strict measurement invariance) and the main conclusions of our study did not differ in comparison to the analyses conducted in the article. Please contact the first author for the detailed results.

Supplemental Material B references

Bentler, P. M., & Chou, C.-P. (1987). Practical issues in structural modeling. *Sociological Methods & Research*, *16*(1), 78-117. https://doi.org/10.1177/0049124187016001004

Brown, T. A. (2015). *Confirmatory factor analysis for applied research* (2nd ed.). Guilford Press.

Byrne, B. M. (2012). *Structural equation modeling with mplus: Basic concepts, applications, and programming*. Routledge.

Cole, D. A., Ciesla, J. A., & Steiger, J. H. (2007). The insidious effects of failing to include design-driven correlated residuals in latent-variable covariance structure analysis. *Psychological Methods*, *12*(4), 381-398. https://doi.org/10.1037/1082-989X.12.4.381
